# How to gauge investor behavior? A comparison of online investor sentiment measures

**DOI:** 10.1007/s42521-021-00038-2

**Published:** 2021-08-07

**Authors:** Daniele Ballinari, Simon Behrendt

**Affiliations:** 1 Faculty of Business and Economics, University of Basel, Peter Merian-Weg 6, 4002 Basel, Switzerland; 2 d-fine GmbH, An der Hauptwache 7, 60313 Frankfurt, Germany

**Keywords:** Investor sentiment, Twitter, StockTwits, Order imbalances, Portfolio returns, G11, G17, G40

## Abstract

Given the increasing interest in and the growing number of publicly available methods to estimate investor sentiment from social media platforms, researchers and practitioners alike are facing one crucial question – which is best to gauge investor sentiment? We compare the performance of daily investor sentiment measures estimated from Twitter and StockTwits short messages by publicly available dictionary and machine learning based methods for a large sample of stocks. To determine their relevance for financial applications, these investor sentiment measures are compared by their effects on the cross-section of stocks (i) within a Fama and MacBeth (J Polit Econ 81:607–636, 1973) regression framework applied to a measure of retail investors’ order imbalances and (ii) by their ability to forecast abnormal returns in a model-free portfolio sorting exercise. Interestingly, we find that investor sentiment measures based on finance-specific dictionaries do not only have a greater impact on retail investors’ order imbalances than measures based on machine learning approaches, but also perform very well compared to the latter in our asset pricing application.

## Introduction

In recent years, it has become increasingly popular among investors to comment on or to share their opinion about companies’ stock market performances and prospects on social media platforms, such as Twitter and StockTwits. While institutional investors have the means to actively monitor stock markets and public news over the trading day, social media platforms constitute an especially valuable channel for retail investors to obtain stock market relevant information (e.g., Chen et al. 2014). The trading activities of the latter, often portrayed as noise traders in the spirit of Kyle (1985) and Black (1986), may in part be influenced by subjective beliefs about future cash flows and investment risks. These subjective beliefs are referred to as investor sentiment in behavioral models along the lines of De Long et al. (1990), which assume two types of investors, namely rational, sentiment-free arbitrageurs and irrational, sentiment-prone noise traders. Based on their erroneous conviction of having unique information about future stock prices, noise traders buy (sell) stocks when feeling bullish (bearish) about a company. In addition, both types of traders face downward-sloping demand curves for risky assets, which leads to an equilibrium in which these random beliefs of noise traders influence prices. More precisely, De Long et al. (1990) predict that a positive sentiment shock leads to an increase in prices and, conversely, a negative sentiment shock to a decrease in prices.

While prior research has disregarded the role of irrational investors, assuming that arbitrageurs would trade against them and keep prices at their fundamental values (Friedman 1953; Fama 1965), behavioral models following De Long et al. (1990) and Shleifer and Vishny (1997) instead suggest that arbitrageurs are likely to be risk-averse, and their willingness to trade against noise traders is limited. The model introduced by De Long et al. (1990), for instance, postulates that arbitrageurs face not only fundamental risks when taking positions against noise traders but also the risk that the beliefs of irrational investors may not reverse to their mean for a prolonged period of time. This implies that noise traders can drive stock prices away from their fundamental values, at least over short time periods, given that the willingness of risk-averse arbitrageurs to bet against them is limited.^1^

Thus, the classical finance theory in which the cross-section of expected returns is affected only by the cross-section of systemic risk in equilibrium has been augmented by these behavioral aspects. To this end, retail investors have been shown empirically to trade excessively in attention-grabbing stocks (Barber and Odean 2007) and in concert with other retail investors (Kumar and Lee 2006; Barber et al. 2009), having a significant impact on stock prices.

Following this line of thought and the initial findings of Antweiler and Frank (2004) and Das and Chen (2007), a vast literature has evolved around the question of how to augment and improve forecasts of financial variables, such as stock returns, volatility, and trading volume, with measures of investor sentiment derived from online sources (for a recent survey, see Nardo et al. 2016).^2^ For example, Sprenger et al. (2014a) obtain good and bad news from Twitter messages related to the S&P 500 and link these news to market movements. Yang et al. (2015) provide further empirical evidence for the existence of a financial community on Twitter and demonstrate that the weighted sentiment of its most influential contributors has significant predictive power for such market movements. Da et al. (2015) use online search queries of sentiment-specific terms to construct a measure of market-wide investor sentiment. Their results are broadly in line with the theories on investor sentiment mentioned above. Concerning individual-level stocks, Sprenger et al. (2014b) find an association between Twitter sentiment and returns as well as the volume of Twitter messages and trading volume. Moreover, making use of stock picks from the CAPS website, Avery et al. (2015) demonstrate that negative stock picks strongly predict future stock price declines.^3^ Other findings point towards a relation between message board posts and contemporaneous returns of underperforming small-cap stocks (Leung and Ton 2015). Recently, some studies have investigated the predictive performance of online investor sentiment measures at intraday frequencies. While Behrendt and Schmidt (2018) show that the economic significance of Twitter sentiment in intraday volatility forecasting applications is negligible, Renault (2017) provides some empirical evidence for sentiment-driven noise trading throughout the trading day using investor sentiment estimated from StockTwits messages.

In light of these empirical findings, which involve different online sources and methods to estimate investor sentiment, both researchers and practitioners alike are still facing one crucial question – how to measure and quantify investor sentiment adequately? As far as textual analysis in finance is concerned, conventional approaches usually involve dictionaries and machine learning techniques (for recent surveys, see Das et al. 2014; Kearney and Liu 2014). The latter are predominantly used when online investor sentiment is estimated from individual messages published on social media platforms, such as Twitter and StockTwits, since dictionaries developed for short messages that also cover financial topics are scarce. By contrast, methods based on dictionaries, such as the Harvard-IV dictionary or the dictionary of Loughran and McDonald (2011), are more often used in the context of textual analysis of traditional news channels. An exception are the dictionaries of Renault (2017), which are tailored to finance-specific short messages on StockTwits. Although dictionaries are usually publicly available and ready to use, this is not the case for most approaches based on machine learning techniques. Lastly, some commercial data vendors offer investor sentiment measures for researchers and practitioners to use. While these commercial measures may increase the reproducibility of findings, they are inherently opaque since the exact way of calculating the respective measure is usually not publicly disclosed.

This paper contributes to the literature in several ways: (i) we estimate daily online investor sentiment from short messages published on Twitter and StockTwits for 360 stocks over a seven years time period from the beginning of 2011 to the end of 2017 with a wide selection of sentiment estimation techniques used in the finance literature, (ii) the performance of the different approaches is compared by means of financial applications, and (iii) we rank and explain the performance of the dictionaries as well as the machine learning approaches in order to provide a guideline for both researchers and practitioners on the basis of field-specific applications. To be more precise, we estimate investor sentiment with five publicly available dictionaries, two open-source and pre-trained neural networks, and two simple machine learning models trained by us on labelled StockTwits data. The dictionaries considered in this paper are the Harvard-IV dictionary, the dictionary of Loughran and McDonald (2011) (hereafter, LM), both short message- and finance-specific dictionaries of Renault (2017) (hereafter, L1 and L2), and the VADER dictionary (Hutto and Gilbert 2014), which is a general dictionary optimized for short messages. The machine learning models used to estimate investor sentiment are the naive Bayes classifier, maximum entropy, the convolutional neural network Deep-MLSA of Deriu et al. (2017), and the long short-term memory neural network DeepMoji of Felbo et al. (2017). Note that the focus of this paper is on publicly available sentiment estimation techniques. For a further comparison of trainable machine learning approaches, we refer to Renault (2019). While some of the prior research has focused on analyses at lower frequencies and over longer time horizons, we follow more recent literature by considering a daily frequency. Moreover, we also make use of the method proposed by Boehmer et al. (2020) for the identification of retail investor trades in the NYSE Trade and Quote (TAQ) database. This allows us to more closely adhere to the above-mentioned theoretical models and to relate the effects of online investor sentiment to order imbalances based on trades conducted by these investors.

Our comparison of the above-mentioned sentiment measures is based on two financial applications that are helpful to study the effect of online investor sentiment on the cross-section of stocks, which is of central importance in both classical and behavioral finance theory (see Baker and Wurgler 2006, 2007, for a discussion): Firstly, we investigate the effect of each sentiment measure on the cross-section of retail investors’ order imbalances within a model framework in the spirit of Fama and MacBeth (1973). This allows us to estimate the direct impact of the sentiment measures on trades initiated by retail investors. Secondly, since asset pricing applications are often of primary interest for researchers and practitioners, we use the sentiment measures in a model-free portfolio sorting exercise and forecast abnormal portfolio returns. Overall, while the performance of the considered sentiment measures varies considerably, we find that the LM dictionary of Loughran and McDonald (2011) and the L2 dictionary of Renault (2017) perform well in terms of their effect on retail investors’ order imbalances and their ability to forecast abnormal portfolio returns. Thus, finance-specific dictionaries perform on par with or even better than state-of-the-art machine learning approaches.

The remainder of the paper is structured as follows: Section 2 describes the different online investor sentiment measures, their calculation, and some instructive descriptive statistics of the data set. The effect of the respective online investor sentiment measures on the cross-section of retail investors’ order imbalances is investigated within a Fama-MacBeth (1973) regression framework in Section 3 and, subsequently, in a model-free portfolio sorting application to forecast abnormal portfolio returns in Section 4. Lastly, Section 5 offers some concluding remarks.

## Online investor sentiment data

### The raw text data

We consider two sources of online text data that are widely used in the finance literature, namely Twitter (e.g., Sprenger et al. 2014a, b; Yang et al. 2015; Bartov et al. 2018; Audrino et al. 2020; Lehrer et al. 2019; Nofer and Hinz 2015; Rao and Srivastava 2014; Ballinari and Behrendt 2020) and StockTwits (e.g., Audrino et al. 2020; Cookson and Niessner 2020; Giannini et al. 2019; Renault 2017; Guégan and Renault 2020; Mahmoudi et al. 2018; Ballinari and Behrendt 2020). Twitter is a social media network with roughly 126 million active daily users where people can share thoughts, ideas, and opinions in the form of short messages consisting of 140 characters.^4^ Similarly, StockTwits also allows users to share 120-character messages with the online community, the difference being that it is specifically tailored towards investors and traders. Focusing on the time period between 2011 and 2017, we analyze 360 companies that are constantly part of the S&P 500 during that time.^5^

Our motivation for focusing on S&P 500 stocks is twofold. Firstly, to accurately compute daily sentiment measures, we want to consider companies mentioned in large amounts of social media messages. For the same reason, Cookson and Niessner (2020) focus on the 100 stocks with the highest posting volume on StockTwits. Secondly, considering the S&P 500 universe makes our analysis conservative in the sense that we rule out the possibility that our results are driven by micro-capitalized stocks.

Messages shared on StockTwits are directly obtained through the StockTwits API, whereas Twitter messages are collected by following the procedure outlined in Hernandez-Suarez et al. (2018). We collect all shared messages from Twitter and StockTwits either mentioning a company’s name or its cashtag (the company’s ticker symbol preceded by the dollar sign, e.g., “$AAPL” for Apple Inc.).^6^ For both data sources, we account for changes in a company’s name or ticker.^7^ In total, we collect 30,520,617 and 9,890,132 relevant short messages from Twitter and StockTwits, respectively.

### Different sentiment estimation techniques

After collecting text data from social media platforms, one faces the challenge of transforming the unstructured text data into a quantitative measure for the latent investor sentiment. The two main approaches used for sentiment analysis in finance are dictionary-based and machine learning-based techniques (Das et al. 2014). Table 1 summarises the approaches that are considered in this study. For each dictionary and machine learning model, the table reports a selection of previous studies that make use of the respective sentiment estimation technique. The primary focus of this paper is publicly available dictionaries and pre-trained machine learning models that researches can directly use. Nevertheless, given their great popularity in the finance literature, we have also included the naive Bayes and maximum entropy classifiers into our analysis, which are trained on our StockTwits data.

**Table 1:** Overview of investor sentiment estimation techniques

Panel A: dictionary based approaches	
Dictionary	Selection of studies using the dictionary
Harvard-IV	Tetlock (2007); Tetlock et al. (2008); Engelberg et al. (2012); Hanley and Hoberg (2010); Engelberg (2008); Renault (2017)
LM	Loughran and McDonald (2011); Engelberg et al. (2012); Garcia (2013); Chen et al. (2014); Liu and McConnell (2013); Dougal et al. (2012); Renault (2017)
VADER	Audrino et al. (2020); García-Medina et al. (2018)
L1 and L2	Renault (2017); Ballinari and Behrendt (2020)

Panel B: machine learning based approaches	
Model	Selection of studies using the model
Naive Bayes	Antweiler and Frank (2004); Rao and Srivastava (2014); Sprenger et al. (2014b); Leung and Ton (2015)
Maximum entropy	Cookson and Niessner (2020); Giannini et al. (2019); Renault (2017)
Deep-MLSA	Audrino et al. (2020); Ballinari and Behrendt (2020)
DeepMoji	Lehrer et al. (2019)

Note: This table lists the dictionaries and machine learning approaches used in this study to estimate investor sentiment from short messages published on Twitter and StockTwits. For each approach, we report a selection of studies that make use of the respective dictionary or machine learning approach

#### Dictionary based approaches

In the finance literature, dictionary-based approaches are the most widely adopted methodologies to gauge the mood and sentiment enclosed in text data. These approaches are based on a list of words associated with a particular sentiment (e.g., positive or negative). One then counts the number of times that words with a particular connotation occur in the analyzed text. In the case of a social media post, for instance, we count the number of positive and negative words used in the message as defined by a specific dictionary. We then categorize the message as being optimistic (or, in the context of finance, bullish) if more words with a positive than a negative connotation are identified. The use of dictionaries for sentiment analysis has several advantages: Firstly, the computational cost of counting positive and negative words is usually low. Secondly, the implementation of a dictionary based approach is relatively simple and transparent. Thirdly, being a computationally feasible and transparent approach, results based on dictionaries are relatively straightforward to reproduce. Lastly, most dictionaries are publicly available. Dictionaries commonly used for sentiment analysis range from very broad and general to field-specific lists of words (often called lexicons).

Initially, the most frequently used dictionary for sentiment analysis in finance has been Harvard-IV, a general-purpose dictionary developed by Harvard University and used in the General Inquirer software.^8^ We refer to Loughran and McDonald (2016) for a more extensive review of studies using this general-purpose dictionary. The Harvard-IV dictionary consists of 2005 negative and 1637 positive words. After applying standard pre-processing methods to the textual data (e.g., tokenizing, transforming words into lower case, and removing stop words), we use the dictionary to capture the tone of social media messages by counting the number of positive and negative words. The sentiment score of a given Twitter or StockTwits short message is then defined as the difference between the share of positive and negative words. As a result, we obtain a sentiment score ranging from −1 (negative) to +1 (positive).

However, the use of general-purpose dictionaries, such as the Harvard-IV dictionary, for sentiment analysis in finance might produce misleading results (Loughran and McDonald 2016; Renault 2017). Almost three-fourths of the words classified as having a negative tone by the Harvard-IV dictionary do not necessarily have a negative connotation in finance-related sentences (for example, “tax”, “cost”, “capital”, and “liability”). Motivated by this issue, Loughran and McDonald (2011) have developed a dictionary consisting of six different word lists (negative, positive, uncertain, litigious, strong modal, and weak modal). The dictionary, often abbreviated as LM, is constructed using a large sample of Form 10-K filings of US companies during the period from 1994 to 2008. After creating a dictionary of words occurring in at least 5% of the filings, Loughran and McDonald (2011) classify each word based on its most likely connotation in a finance context. In our analysis, we consider only the positive and negative word lists consisting of 354 and 2,355 words, respectively. The dictionary and Python implementations for textual sentiment analysis are available at the software repository for accounting and finance of the University of Notre Dame.^9^ Again, the sentiment of a given social media message is calculated as the difference between the share of positive and negative words occurring in the pre-processed text data.

Since the LM dictionary is constructed using words occurring in 10-K filings, the semantic connotations of typical expressions used on social media platforms are not necessarily captured. Emoticons (e.g., a smiling face), abbreviations (e.g., “LOL” stands for “laughing out loud”), or slang (e.g., “nah” or “meh”), which most likely have some sentiment connotation, are not covered by the Harvard-IV and the LM dictionaries. VADER (Valence Aware Dictionary and sEntiment Reasoner), the dictionary and rule-based approach introduced by Hutto and Gilbert (2014), is specially constructed to capture the sentiment of short and informal text messages, such as those published on social media platforms. In a first step, a dictionary is created by combining word lists from existing general-purpose dictionaries and common expressions occurring in social media messages (e.g., emoticons and abbreviations). The semantic connotation of each of the roughly 7500 words and expressions is obtained by averaging the opinion of ten independent human raters. Contrary to the previous two dictionaries, the VADER word list does not only classify a word as being positive or negative, but also defines the intensity of a word’s sentiment. In a second step, Hutto and Gilbert (2014) define a rule-based model that increases or decreases the sentiment intensity of a text based on five grammatical and syntactical heuristics (e.g., punctuation, upper case letters). For our analysis, sentiment scores based on this dictionary and rule-based approach are obtained by processing the social media data with the publicly available implementation of VADER.^10^

While the methodology introduced by Hutto and Gilbert (2014) accounts for the short and informal structure of textual data obtained from social media platforms, it is still based on a general-purpose dictionary and thus might not classify words with a finance-specific meaning correctly (e.g., “liability” has a negative connotation in the VADER dictionary). In a recent study, Renault (2017) proposes two dictionaries specifically designed to capture the sentiment in finance-related social media short messages. The dictionaries are constructed based on messages shared on StockTwits and by exploiting a feature of this social media platform introduced in 2012 that allows users to “tag” their short messages as being either bullish (i.e., positive) or bearish (i.e., negative). The first dictionary, hereafter referred to as Renault L1, is constructed by selecting all uni-grams (1 word) and bi-grams (2 subsequent words) appearing at least 75 times in a sample of 750,000 StockTwits messages.^11^ The semantic connotation of each word is then defined as the difference between the share of appearances in bullish and bearish messages. The dictionary is refined by only considering the 20% most positive and the 20% most negative words (in total 8000 items). Due to anomalies identified in the data-driven dictionary (e.g., the word “commodity” has a negative connotation as a result of the decline in commodity prices during the sample period), Renault (2017) proposes a second dictionary, hereafter referred to as Renault L2, constructed by manually classifying the uni-grams and bi-grams as positive, neutral, or negative. The Renault L2 dictionary consists of 543 positive and 768 negative terms. In our analysis, we compute the sentiment of messages from Twitter and StockTwits using both the Renault L1 and L2 dictionaries.^12^ The textual data are pre-processed by following the approach outlined in Renault (2017), and the sentiment connotation of a given message is defined as the difference between the share of positive and negative terms.

To sum up, we compare five publicly available dictionaries being either of general purpose or specific to a particular field (social media platforms, finance-related text). Table 2 illustrates the commonalities and differences among the five considered dictionaries. More precisely, the table reports the number of shared terms between pairs of dictionaries (the diagonal elements show the total number of words in each dictionary). In parentheses below the number of common terms, we report the share of words to which two dictionaries assign the same sentiment connotation. Except for the comparison of the Renault L1 dictionary with the Harvard-IV and the VADER dictionaries, the share of words with the same sentiment direction between two dictionaries is relatively high, ranging from 93.2 to 99.9%. Table 2 highlights two main differences regarding the five considered dictionaries: Firstly, the number of common terms between the field-specific and general-purpose dictionaries is low. For example, less than one-fifth of the words in the LM dictionary are also part of the Harvard-IV dictionary. Secondly, among the field-specific dictionaries, the number of shared terms between the LM dictionary and the word lists specially constructed for finance-related short messages is also low.

**Table 2:** Comparison of shared terms among dictionaries

	Harvard-IV	LM	L1	L2	VADER
Harvard-IV	3642	597	312	235	1291
	(100%)	(97.3%)	(75.6%)	(93.2%)	(97.7%)
LM		2709	191	217	870
		(100%)	(96.9%)	(99.5%)	(97.6%)
L1			8000	805	418
			(100%)	(99.9%)	(81.6%)
L2				1311	373
				(100%)	(97.3%)
VADER					7517
					(100%)

Note: This table summarizes commonalities and differences among five publicly available sentiment dictionaries, i.e., Harvard-IV, LM (Loughran and McDonald 2011), the two dictionaries introduced by Renault (2017) (L1 and L2), and VADER (Hutto and Gilbert 2014). More precisely, the table shows the number of common terms occurring in two dictionaries. The diagonal elements report the total number of words in each dictionary and the off-diagonal elements report the number of shared terms between two dictionaries. The share of words having the same sentiment connotation (positive or negative) in both dictionaries is reported in parentheses below the respective number of common terms

#### Machine learning techniques

With increasing computational power, the popularity of machine learning algorithms in the context of sentiment classification has increased as well. The underlying idea of these techniques is to train a model to predict the sentiment of a text given a set of features (predictors). The main steps for implementing such a methodology are (i) the definition of the predictors (often referred to as feature engineering), (ii) estimating the relevant parameters (training), and (iii) evaluating the model’s accuracy (testing). Compared to dictionary-based approaches, the use of machine learning techniques has some advantages: Firstly, these models can better capture the complex structure of text data, whereas the dictionaries discussed above rely on the assumption that words (or, at most, bi-grams) in a sentence are independent (i.e., their ordering does not matter). Secondly, instead of selecting words and determining their connotation, machine learning techniques are more flexible in choosing relevant features. However, there are also some drawbacks: Firstly, the classification accuracy of the model highly depends on the quantity and quality of the training data. This implies that a large amount of pre-classified text data is necessary to train and test a model properly (Renault 2017). Furthermore, the predictions made by these models are generally nontransparent and challenging to comprehend. For a comparison of different machine learning classifiers for social media messages about finance, we refer to Renault (2019).

Two of the most popular machine learning approaches used for sentiment classification are the naive Bayes classifier and maximum entropy models (e.g., Cookson and Niessner 2020; Giannini *et al.* 2019).^13^ Recently, researchers have also started to rely more frequently on neural networks. Mahmoudi et al. (2018), among others, train convolutional and recurrent neural networks to classify StockTwits messages. Unfortunately, the number of pre-trained sentiment classification models that are publicly available is quite small, especially considering field-specific models. To the best of our knowledge, there exist no publicly available sentiment classification algorithms trained specially for finance-related text data.

One of the only publicly available and trained machine learning approaches for sentiment classification is the (deep) convolutional neural network proposed by Deriu et al. (2017), hereafter referred to as Deep-MLSA. Several facts motivate our choice of this model: Firstly, as already mentioned, the authors have made a pre-trained Python implementation of their model publicly available.^14^ Secondly, the model has been specially trained for classifying social media short messages. Furthermore, having won the message polarity classification task “Sentiment Analysis in Twitter” at the 2016 SemEval competition, this technique can be considered as one of the best performing sentiment classification approaches for social media short messages currently available. For a detailed description of the model and training procedure, we refer to Deriu et al. (2017).

For the sake of completeness, we also consider three other machine learning approaches used in the finance literature. More precisely, we consider the long short-term memory neural network introduced by Felbo et al. (2017), a naive Bayes classifier, and a maximum entropy model. As mentioned above, the naive Bayes and maximum entropy models have been trained on labelled StockTwits data since there exist no pre-trained models that are publicly available.

The (deep) neural network developed by Felbo et al. (2017), hereafter referred to as DeepMoji, is trained to predict the emoticons associated with a tweet. The authors train a long short-term memory network to predict the probability that one of 64 considered emoticons occurs in a given social media post. For a detailed description of the model and the pre-training, we refer to Felbo et al. (2017). There exists a Python implementation of the pre-trained DeepMoji model that is publicly available.^15^ However, to use this model for a binary classification task (e.g., bullish and bearish short messages), it is necessary to further train and fine-tune the neural network. We do this by following the approach of Renault (2017) and Mahmoudi et al. (2018), i.e., using the self-reported labels associated with StockTwits messages. To be more precise, the training set contains all 241,591 labeled messages published about the 360 companies considered in this study between Jun 1, 2013 and Aug 31, 2014.^16^ Due to the larger proportion of messages tagged as being “bullish”, we obtain a balanced training set by undersampling the positive messages. We retain 30% of the training data for validating the model. The so constructed training and validation data sets are then used to train and fine-tune the DeepMoji model of Felbo et al. (2017).^17^

The naive Bayes and maximum entropy approaches are trained with the same data set of labeled StockTwits messages described in the previous paragraph. We apply standard cleaning procedures to the textual data, i.e., we turn all words into lowercase, remove stop-words and punctuation, shorten repeated characters (e.g., “allllll” becomes “all”), apply the wordnet lemmatizer to each token, and replace URLs, user-names, company names, cashtags, and numbers with corresponding tags (e.g., “tag_username” or “tag_url”).^18^ We adopt a bag-of-words representation for the cleaned text data. Following the results documented in Renault (2019), we consider both uni- and bi-grams. The bag-of-words representation of the tweets is stored in a term frequency-inverse document frequency (TF-IDF) document-term matrix.^19^ We then train the naive Bayes and maximum entropy classifiers using the so defined matrix of predictors (see Hastie et al. 2009, for a general description of the models).

### Aggregation to a daily investor sentiment measure

After classifying social media short messages as having either a positive or negative sentiment connotation, one usually needs to aggregate the unevenly spaced sentiment scores to obtain an evenly spaced time series at a lower frequency. In this paper, we focus on the construction of daily sentiment measures. We define a day to start at 16:00 Eastern Time of the previous trading day and end at 16:00 Eastern Time of the current day. In the finance literature, different aggregation schemes have been suggested. Renault (2017, 2019) and Cookson and Niessner (2020), among others, aggregate the sentiment scores of StockTwits short messages to a lower frequency with a simple empirical average. In our case, for company *i* on day *t*, denoting the empirical average by *A*_*i*,* t*_, this amounts to:

1 $$\begin{aligned} A_{i,t} = \frac{1}{N_{i,t}} \sum _{t_n} S_{i,t_n}, \end{aligned}$$

where $N_{i,t}$ refers to the total number of short messages published on a social media platform about company *i* on day *t*, and $S_{i,t_n}$ is the sentiment score at intraday time $t_n$, with $n = 1, 2, \ldots , N$, assigned to a short message ranging from −1 (negative sentiment) to +1 (positive sentiment). By contrast, Antweiler and Frank (2004) propose a so-called bullishness measure, denoted here by $B_{i,t}$ and defined as:

2 $$\begin{aligned} B_{i,t} = \log \left( \frac{1 + N_{i,t}^{pos}}{1 + N_{i,t}^{neg}} \right) , \end{aligned}$$

where $\log (\cdot )$ stands for the natural logarithm, $N_{i,t}^{pos}$ is the number of messages classified as being positive, and $N_{i,t}^{neg}$ the number of messages classified as being negative.

We conduct our analysis for both aggregation schemes but only report those results obtained with the bullishness measure since the results show some discrepancies between aggregation schemes and seem more plausible for the bullishness measure. The findings obtained with the average aggregation scheme are available from the authors upon request. The reason behind the discrepancies in the results most likely stems from the fact that the measure proposed by Antweiler and Frank (2004) also takes into account the volume of messages posted over a given day. To be more precise, the bullish sentiment can be approximated by $B_{i,t} \approx \log (1+N_{i,t}^{pos} + N_{i,t}^{neg}) \left( N_{i,t}^{pos} - N_{i,t}^{neg} \right) /\left( N_{i,t}^{pos} + N_{i,t}^{neg} \right)$. Consider, for example, that over a given day only one message about Apple Inc. is posted and classified as being positive, while on another day 1,000 messages mentioning Apple Inc. are published and all are classified as being positive. The average sentiment is the same for both days, i.e., $A_{i,t} = 1$. However, the bullish sentiment for the first day is $B_{i,t} = \log (2) \approx 0.69$, and for the second day $B_{i,t} = \log (1001) \approx 6.91$. As such, the aggregation approach proposed by Antweiler and Frank (2004) considers not only the sentiment but also the intensity of investors’ attention, which has been shown to have a significant impact on future stock returns (see, among others, Barber and Odean 2007; Da et al. 2011). Note that even after aggregating the estimated sentiment of social media short messages to the daily frequency, it is still possible that no messages about a company are shared on Twitter or StockTwits on a given day. For those days, we make the simplifying assumption that investors’ sentiment remains unchanged until the next message is published, i.e., we replace the missing daily bullish sentiment with the most recent observation.^20^

**Table 3:** Correlations of daily bullish sentiment across sentiment measures

Panel A: Twitter									
	(1)	(2)	(3)	(4)	(5)	(6)	(7)	(8)	(9)
Harvard-IV (1)	1.00	0.25	0.20	0.26	0.37	0.11	0.10	0.06	0.18
LM (2)		1.00	0.20	0.31	0.39	0.17	0.17	0.20	0.22
L1 (3)			1.00	0.50	0.25	0.30	0.31	0.05	0.38
L2 (4)				1.00	0.33	0.34	0.34	0.11	0.41
VADER (5)					1.00	0.24	0.21	0.19	0.33
Naive Bayes (6)						1.00	0.74	0.20	0.45
Max. entropy (7)							1.00	0.16	0.45
Deep-MLSA (8)								1.00	0.15
DeepMoji (9)									1.00

Panel B: StockTwits									
	(1)	(2)	(3)	(4)	(5)	(6)	(7)	(8)	(9)
Harvard-IV (1)	1.00	0.28	0.19	0.32	0.41	0.20	0.24	0.15	0.23
LM (2)		1.00	0.22	0.31	0.37	0.16	0.18	0.23	0.21
L1 (3)			1.00	0.55	0.24	0.35	0.39	0.13	0.38
L2 (4)				1.00	0.36	0.48	0.52	0.18	0.53
VADER (5)					1.00	0.24	0.25	0.22	0.27
Naive Bayes (6)						1.00	0.76	0.14	0.57
Max. entropy (7)							1.00	0.14	0.57
Deep-MLSA (8)								1.00	0.15
DeepMoji (9)									1.00

Panel C: correlation between Twitter and StockTwits									
	(1)	(2)	(3)	(4)	(5)	(6)	(7)	(8)	(9)
StockTwits-Twitter	0.26	0.32	0.27	0.33	0.31	0.22	0.22	0.25	0.29

This table reports correlations between daily bullish sentiment scores estimated from short messages published on Twitter and StockTwits based on different approaches (dictionary based and machine learning techniques). Panel A and B report correlations between daily bullish sentiment scores estimated from Twitter and StockTwits short messages, respectively. Panel C reports correlations between daily bullish sentiment scores estimated from Twitter short messages with those estimated from StockTwits short messages

Table 3 reports correlations between the estimated bullish sentiment for all considered sentiment measures and data sources, pooled over companies and days. More precisely, Panel A and B report the correlations between daily bullish sentiment scores obtained by the five dictionaries and the four machine learning models as estimated from short messages posted on Twitter and StockTwits, respectively. Panel C reports for each sentiment measure the correlation between the bullish sentiment obtained from Twitter short messages and the bullish sentiment obtained from StockTwits short messages. Noteworthy is the fact that bullish sentiment obtained by the LM dictionary is most highly correlated with that obtained by the VADER rule-based approach. Moreover, we find that when using Twitter data, the bullish sentiment obtained by the Deep-MLSA model has a very low correlation with the daily sentiment measures obtained by dictionary based approaches. Moreover, the correlations between the two dictionaries proposed by Renault (2017), the naive Bayes, maximum entropy, and DeepMoji neural network are relatively high. This may not surprise since all five sentiment estimation approaches are estimated and trained on StockTwits data. The results presented in Panel C of Table 3 show that the correlation between sentiment measures obtained from Twitter and StockTwits messages is between 0.2 and 0.3. The LM, L1, L2, and VADER dictionaries appear to produce the most “consistent” bullish sentiment signal across the two social media platforms.

Table 4 reports summary statistics for daily bullish online investor sentiment derived from Twitter (Panel A) and StockTwits (Panel B), again pooled over companies and days. For each of the six sentiment estimation approaches, the table reports the mean daily bullish sentiment and its 1, 10, 25, 50, 75, 90, and 99%-quantiles. The table uncovers two essential features of the sentiment scores: Firstly, we note that the average daily bullish sentiment is positive and the median non-negative, regardless of the data source or sentiment estimation technique being considered. The proportion of days with negative daily bullish sentiment is rather low. Especially the VADER dictionary and the Deep-MLSA model classify very few short messages as having a negative investor sentiment. Secondly, a considerable number of days have a neutral investor sentiment, i.e., the bullish sentiment is zero. In particular, when sentiment is estimated with the LM dictionary and the Deep-MLSA model. This effect is more pronounced when investor sentiment is estimated from short messages published on StockTwits. For instance, when applying the LM dictionary and the Deep-MLSA model to StockTwits messages, 59.9 and 73.2% of the days in our sample have a neutral sentiment, respectively.

**Table 4:** Summary statistics for daily bullish sentiment

Panel A: Twitter								
	$Q_{1\%}$	$Q_{10\%}$	$Q_{25\%}$	Median	Mean	$Q_{75\%}$	$Q_{90\%}$	$Q_{99\%}$
Harvard-IV	−1.61	−0.69	0.00	0.56	0.51	1.10	1.61	2.56
LM	−2.14	−1.10	−0.41	0.00	0.05	0.69	1.10	2.14
L1	−1.59	−0.69	0.00	0.51	0.49	1.10	1.56	2.48
L2	−1.79	−0.69	0.00	0.41	0.41	1.10	1.61	2.56
VADER	−1.25	0.00	0.26	0.86	0.88	1.42	1.98	2.94
Naive Bayes	−1.20	−0.19	0.41	0.88	0.85	1.39	1.83	2.74
Max. entropy	−1.39	−0.41	0.12	0.69	0.62	1.10	1.61	2.48
Deep-MLSA	−1.95	−0.69	0.00	0.00	0.15	0.69	1.10	2.30
DeepMoji	−1.10	0.00	0.58	1.10	1.06	1.61	2.08	3.00

Panel B: StockTwits								
	$Q_{1\%}$	$Q_{10\%}$	$Q_{25\%}$	Median	Mean	$Q_{75\%}$	$Q_{90\%}$	$Q_{99\%}$
Harvard-IV	−1.39	-0.69	0.00	0.00	0.24	0.69	1.10	1.79
LM	−1.39	−0.69	0.00	0.00	0.02	0.00	0.69	1.39
L1	−1.39	−0.69	0.00	0.41	0.38	0.77	1.39	2.20
L2	−1.39	−0.69	0.00	0.29	0.32	0.69	1.25	2.20
VADER	−1.10	−0.29	0.00	0.41	0.44	0.69	1.39	2.08
Naive Bayes	−1.10	−0.69	0.00	0.69	0.59	1.10	1.39	2.30
Max. entropy	−1.39	−0.69	0.00	0.69	0.49	1.10	1.39	2.20
Deep-MLSA	−1.20	−0.59	0.00	0.00	0.06	0.00	0.69	1.39
DeepMoji	−1.10	−0.69	0.13	0.69	0.63	1.10	1.61	2.40

Note: This table reports summary statistics for daily bullish sentiment constructed from Twitter (Panel A) and StockTwits (Panel B) data. More precisely, for each of the nine sentiment estimation approaches, the table reports the 1, 10, 25, 50, 75, 90, and 99%-quantiles as well as the mean daily bullish sentiment

### Filtering tweets and companies

Following the data collection approach described previously (see Section 2.1), all messages that mention a company’s name and/or cashtag are considered for the construction of the daily bullishness score. Since a single message potentially mentions more than just one company, it becomes difficult to attribute the negative or positive connotation of a social media post to a specific company’s stock. Thus, in addition to our baseline data collection approach, we also consider a more conservative selection of short messages and following, among others, Cookson and Niessner (2020), we consider only posts on Twitter and StockTwits that mention a unique cashtag. Table 5 reports the correlations across sentiment scores. In comparison with Table 3, the correlation coefficients for many of the nine sentiment estimation techniques have increased.

**Table 5:** Correlations of daily bullish sentiment across sentiment measures (unique cashtags)

Panel A: Twitter									
	(1)	(2)	(3)	(4)	(5)	(6)	(7)	(8)	(9)
Harvard-IV (1)	1.00	0.26	0.24	0.29	0.41	0.12	0.11	0.12	0.23
LM (2)		1.00	0.21	0.31	0.33	0.11	0.12	0.20	0.17
L1 (3)			1.00	0.53	0.30	0.35	0.34	0.11	0.45
L2 (4)				1.00	0.32	0.38	0.38	0.16	0.47
VADER (5)					1.00	0.20	0.18	0.16	0.30
Naive Bayes (6)						1.00	0.73	0.08	0.46
Max. entropy (7)							1.00	0.08	0.45
Deep-MLSA (8)								1.00	0.10
DeepMoji (9)									1.00

Panel B: StockTwits									
	(1)	(2)	(3)	(4)	(5)	(6)	(7)	(8)	(9)
Harvard-IV (1)	1.00	0.28	0.21	0.33	0.40	0.18	0.18	0.14	0.21
LM (2)		1.00	0.20	0.27	0.34	0.16	0.17	0.25	0.20
L1 (3)			1.00	0.52	0.21	0.35	0.37	0.15	0.38
L2 (4)				1.00	0.31	0.48	0.53	0.17	0.54
VADER (5)					1.00	0.21	0.20	0.19	0.22
Naive Bayes (6)						1.00	0.81	0.12	0.55
Max. entropy (7)							1.00	0.13	0.60
Deep-MLSA (8)								1.00	0.13
DeepMoji (9)									1.00

Panel C: correlation between Twitter and StockTwits									
	(1)	(2)	(3)	(4)	(5)	(6)	(7)	(8)	(9)
StockTwits-Twitter	0.28	0.32	0.25	0.32	0.33	0.20	0.20	0.26	0.30

Note: This table reports correlations between daily bullish sentiment scores estimated from short messages that contain a unique cashtag published on Twitter and StockTwits based on different approaches (dictionary-based and machine learning techniques). Panel A and B report correlations between daily bullish sentiment scores estimated from Twitter and StockTwits short messages, respectively. Panel C reports correlations between daily bullish sentiment scores estimated from Twitter short messages with those estimated from StockTwits short messages

## Investor sentiment and retail investors’ order imbalance

Theoretical models of investor sentiment in the context of financial markets assume that there exist two types of investors, namely irrational, sentiment-prone noise traders and rational, sentiment-free arbitrageurs (see, among others, De Long et al. 1990). The former have random beliefs, i.e., not necessarily related to fundamental values, about future cash-flows and dividends. Based on their erroneous conviction of having unique information about future stock prices, noise traders buy (sell) stocks when feeling bullish (bearish) about a company. We therefore expect to observe a positive relation between a given measure of investor sentiment and the future short-term order imbalance of retail investors, i.e., the difference between the volume of buy and sell transactions initiated by retail investors. In other words, when the sentiment of messages published on social media platforms is positive, we expect retail investors to initiate more buy transactions than sell transactions.

We follow the approach suggested by Boehmer et al. (2020) and identify all high-frequency transactions obtained from the TAQ database with exchange code “D” and a price just below (above) a rounded penny as retail initiated buy (sell) transactions. Let $VB_{i,t}$ and $VS_{i,t}$ denote the buy and sell trading volume of retail investors for stock *i* on day *t*, respectively. Retail investors’ order imbalance for stock *i* on day *t* is then defined as:

3 $$\begin{aligned} OI_{i,t} = \frac{VB_{i,t} - VS_{i,t}}{VB_{i,t} + VS_{i,t}}. \end{aligned}$$

Following, among others, Loughran and McDonald (2011) and Da et al. (2011), we consider a Fama-MacBeth (1973) cross-sectional regression framework. Therefore, for each trading day, we regress retail investors’ daily order imbalances on the previous day’s bullish sentiment.^21^ Note that the order imbalance of retail investors defined in Equation (3) is bounded between −1 and +1. To avoid imposing parameter restrictions, we consider the Fisher-transformed order imbalance, which is denoted as $\widetilde{OI}_{i,t} = 0.5\log (1+OI_{i,t}) - 0.5\log (1-OI_{i,t})$. Moreover, we include several control variables in the cross-sectional regression framework. In the spirit of Fama and French (1993) and Carhart (1997), we control for lagged (log) market capitalization, market-to-book ratio, and returns. In addition, we also control for lagged retail investors’ order imbalance, the abnormal news volume defined as the natural logarithm of the ratio between the news volume and its average over the previous 21 days, and the lagged daily realized volatility.^22^ Relevant data are obtained from the Center for Research in Security Prices (CRSP), Compustat, RavenPack News Analytics, and the TAQ database. For each trading day, we run the following cross-sectional regression:

4 $$\begin{aligned} \widetilde{OI}_{i,t+h} = \alpha _t + \beta _t B_{i,t} + \theta _t' X_{i,t} + \varepsilon _{i,t+1}, \qquad \text {for } h = 1, \dots , 4, \end{aligned}$$

where $\widetilde{OI}_{i,t}$ is the Fisher-transformed retail investor order imbalance of company *i* on trading day *t*, $B_{i,t}$ is the daily bullish sentiment measure, and $X_{i,t}$ is the vector of the above-mentioned control variables. All covariates are standardized such that their coefficients can be interpreted as the effect of a one standard deviation change in the respective variable. The daily regression coefficients are then averaged over time, and Newey-West (1987) standard errors are used to construct *t*-statistics. Following Da et al. (2011), we include only the first lag of bullish sentiment in the cross-sectional regression. However, in the empirical analysis below, we also vary the forecasting horizon. By doing so, we implicitly account for sentiment effects at longer lags.

The regression results are reported in Table 6. Panel A and B report the average daily cross-sectional regression coefficients obtained for the nine different sentiment measures using Twitter and StockTwits data, respectively.^23^ The table reports the average of the estimated regression coefficients for four different forecasting horizons. As mentioned previously, the results presented in theoretical and empirical studies suggest that an increase in retail investors’ sentiment, i.e., noise traders feeling more optimistic about a company, has a positive effect on their order imbalance, at least in the short-term (see, among others, De Long et al. 1990; Tetlock 2007; Chen et al. 2014).

**Table 6:** Fama-MacBeth (1973) regression coefficients for daily bullish sentiment

Panel A: Twitter				
	$t+1$	$t+2$	$t+3$	$t+4$
Harvard-IV	**0.0015**	**0.0016**	**0.0016**	0.0005
	(2.02)	(2.30)	(2.27)	(0.77)
LM	**0.0031**	**0.0027**	**0.0024**	**0.0026**
	(4.30)	(3.90)	(3.50)	(3.60)
L1	**0.0018**	0.0013	0.0003	0.0002
	(2.43)	(1.88)	(0.37)	(0.33)
L2	**0.0035**	**0.0041**	**0.0024**	**0.0018**
	(4.94)	(5.69)	(3.01)	(2.33)
VADER	**0.0015**	0.0004	0.0008	0.0007
	(2.00)	(0.60)	(1.17)	(0.92)
Naive Bayes	**0.0036**	**0.0036**	**0.0020**	**0.0020**
	(4.84)	(4.85)	(2.46)	(2.55)
Max. entropy	**0.0031**	**0.0036**	**0.0022**	**0.0016**
	(3.85)	(4.93)	(2.73)	(1.97)
Deep-MLSA	0.0007	0.0006	0.0008	0.0004
	(1.11)	(0.86)	(1.20)	(0.59)
DeepMoji	**0.0016**	0.0011	0.0004	0.0006
	(2.16)	(1.65)	(0.50)	(0.75)

Panel B: StockTwits				
	$t+1$	$t+2$	$t+3$	$t+4$
Harvard-IV	**0.0015**	0.0009	**0.0017**	0.0009
	(2.09)	(1.21)	(2.27)	(1.21)
LM	**0.0038**	**0.0026**	**0.0016**	**0.0019**
	(5.60)	(3.81)	(2.25)	(2.76)
L1	0.0013	0.0008	0.0006	−0.0003
	(1.76)	(1.17)	(0.70)	(−0.45)
L2	**0.0022**	**0.0022**	0.0014	0.0014
	(3.03)	(3.03)	(1.89)	(1.93)
VADER	**0.0014**	0.0014	**0.0017**	0.0013
	(2.06)	(1.95)	(2.19)	(1.61)
Naive Bayes	0.0010	0.0015	0.0014	0.0003
	(1.43)	(1.94)	(1.76)	(0.37)
Max. entropy	0.0006	0.0008	0.0006	0.0001
	(0.89)	(1.14)	(0.81)	(0.14)
Deep-MLSA	−0.0009	−0.0004	−0.0009	−0.0010
	(−1.45)	(−0.63)	(−1.46)	(−1.58)
DeepMoji	0.0012	0.0010	**0.0016**	0.0006
	(1.55)	(1.28)	(2.01)	(0.75)

Note: The table reports average cross-sectional regression coefficients (see Fama and MacBeth 1973) for daily bullish investor sentiment estimated from short messages published on Twitter (Panel A) and StockTwits (Panel B). The rows refer to the respective investor sentiment measure. The columns represent the dependent variable, being the *h*-day ahead retail investors’ order imbalance, for $h = 1, \dots , 4$. Newey-West (1987) standard errors are used to construct *t*-statistics, which are reported in parentheses below the respective coefficient estimate. All covariates are standardized such that the reported parameters can be interpreted as the effect of a one standard deviation change in the respective variable

To compare the different sentiment measures, we first investigate whether the bullish sentiment has a (significant) positive effect on the 1-day ahead retail investor’s order imbalance and, subsequently, compare the magnitude of this relation. From the first column in Table 6, we observe that, except for the bullish sentiment measure obtained from StockTwits messages and estimated with Deep-MLSA, all regression coefficients are indeed positive. More interesting are the differing magnitudes of the coefficients. For both Twitter and StockTwits data, we obtain the smallest regression coefficients for the sentiment measure based on Deep-MLSA. For Twitter data, the largest impact is observed for the naive Bayes classifier, the L2 dictionary, the LM dictionary, and the maximum entropy classifier. Concerning the StockTwits data, we observe the largest impact for the LM dictionary, followed by the L2 dictionary.

To asses whether these discrepancies are statistically significant, we report *t*-statistics for the pairwise differences between sentiment coefficients obtained from the nine estimation methods in Table 7. Panel A and B report *t*-statistics for the difference between the coefficients obtained with the estimation method reported in the rows with that reported in the columns for messages shared on Twitter and StockTwits, respectively. More precisely, for each data source and each pair of sentiment estimation approach, we construct a time series of differences in the cross-sectional estimates of the sentiment coefficients and compute *t*-statistics to test whether the average difference equals zero (using Newey-West (1987) standard errors). Differences which are statistically significant at the 5% level are highlighted by boldfaced numbers.

**Table 7:** Differences in Fama-MacBeth (1973) regression coefficients for daily bullish sentiment

Panel A: Twitter									
	(1)	(2)	(3)	(4)	(5)	(6)	(7)	(8)	(9)
Harvard-IV (1)	–	**−2.14**	−0.32	**−2.36**	−0.06	**−2.15**	−1.62	0.71	−0.15
LM (2)		–	1.53	−0.54	**2.01**	−0.54	0.01	**2.40**	1.78
L1 (3)			–	**−2.33**	0.25	**−2.14**	−1.39	1.08	0.19
L2 (4)				–	**2.37**	−0.11	0.50	**2.94**	**2.51**
VADER (5)					–	**−2.19**	−1.59	0.82	−0.10
Naive Bayes (6)						–	1.01	**2.87**	**2.50**
Max. entropy (7)							–	**2.29**	1.88
Deep-MLSA (8)								–	−0.85
DeepMoji (9)									–

Panel B: StockTwits									
	(1)	(2)	(3)	(4)	(5)	(6)	(7)	(8)	(9)
Harvard-IV (1)	–	**−3.02**	0.17	−0.91	0.02	0.59	0.97	**2.47**	0.27
LM (2)		–	**2.84**	**2.00**	**2.91**	**3.43**	**3.66**	**5.46**	**2.95**
L1 (3)			–	−1.19	−0.16	0.46	0.85	**2.47**	0.10
L2 (4)				–	0.97	1.89	**2.48**	**3.40**	1.44
VADER (5)					–	0.57	0.92	**2.73**	0.26
Naive Bayes (6)						–	0.59	**2.12**	−0.39
Max. entropy (7)							–	1.74	−0.79
Deep-MLSA (8)								–	**−2.24**
DeepMoji (9)									–

Note: The table reports *t*-statistics for the difference in the average regression coefficients for daily bullish investor sentiment estimated from short messages published on Twitter (Panel A) and StockTwits (Panel B). The dependent variable of the cross-sectional regressions is the 1-day ahead retail investors’ order imbalance. More precisely, we take the difference between coefficients obtained from methodologies reported in the rows with those reported in columns. The *t*-statistics are constructed using Newey-West (1987) standard errors. Differences which are statistically significant at the 5% level are highlighted by boldfaced numbers

Particularly notable are the results for the L2 and LM dictionaries. For messages published on Twitter, we observe that when estimating sentiment with the L2 dictionary the impact of bullish sentiment on $\widetilde{OI}_{i,t+1}$ is statistically larger compared to the Harvard-IV dictionary, the L1 dictionary, VADER, and the two neural networks Deep-MLSA and DeepMoji. Similarly, the impact of Twitter bullish sentiment estimated with the LM dictionary on $\widetilde{OI}_{i,t+1}$ is statistically larger than that of bullish sentiment estimated with the Harvard-IV dictionary, VADER, or Deep-MLSA. For StockTwits messages, bullish sentiment estimated with the L2 dictionary has a significantly larger effect on $\widetilde{OI}_{i,t+1}$ compared to the maximum entropy classifier and Deep-MLSA. The effect of StockTwits bullish sentiment estimated with the LM dictionary on $\widetilde{OI}_{i,t+1}$ is significantly larger than for all other sentiment measures.

In columns 2 through 4 of Table 6, we report the average cross-sectional regression coefficients of daily bullish sentiment for longer horizons. For the sentiment estimation techniques that perform well at the 1-day horizon, the relation remains positive and statistically significant also at longer horizons. In general, however, we observe that the positive relation between bullish sentiment and future order imbalances decreases in magnitude. This result suggests that the two social media platforms considered in this paper are particularly well suited to capture the short-term sentiment of retail investors.

The regression results obtained when estimating retail investors’ sentiment using only social media messages that mention a unique cashtag are reported in Table 8. The corresponding *t*-statistics for the pairwise differences between sentiment coefficients obtained from the nine estimation methods are reported in Table 9. Concerning measures estimated with Twitter data, the effect of bullish sentiment on future retail investors’ order imbalances is smaller compared to the regression results reported in Table 6. Sentiment measures estimated with StockTwits data become instead more informative for future $\widetilde{OI}_{i,t+1}$ when filtering the data. The reason for these changes might be attributed to the fact that the use of cashtags to identify a company when sharing a message on social media is more common on StockTwits than on Twitter. When removing all messages that do not mention a unique cashtag, the number of messages in our sample is reduced by 62% for Twitter and 36% for StockTwits. As such, our filtering approach might remove messages shared on Twitter that contain valuable information about investors’ sentiment, even though they are not mentioning a company’s cashtag. Nevertheless, the results reported in Tables 8 and 9 confirm our previous finding. The L2 and LM dictionaries are overall associated with the largest impact on future order imbalances of retail investors.

**Table 8:** Fama-MacBeth (1973) regression coefficients for daily bullish sentiment (unique cashtags)

Panel A: Twitter				
	$t+1$	$t+2$	$t+3$	$t+4$
Harvard-IV	0.0010	0.0004	0.0003	0.0002
	(1.49)	(0.56)	(0.51)	(0.32)
LM	**0.0028**	**0.0014**	0.0012	0.0011
	(4.22)	(2.06)	(1.68)	(1.53)
L1	0.0014	0.0005	−0.0003	0.0003
	(1.83)	(0.63)	(−0.41)	(0.47)
L2	**0.0027**	**0.0025**	**0.0019**	**0.0017**
	(3.62)	(3.18)	(2.55)	(2.26)
VADER	0.0011	0.0007	0.0011	0.0007
	(1.50)	(1.00)	(1.53)	(0.99)
Naive Bayes	**0.0031**	**0.0021**	0.0006	0.0015
	(4.29)	(2.76)	(0.71)	(1.77)
Max. entropy	**0.0025**	**0.0015**	0.0006	0.0006
	(3.31)	(2.01)	(0.78)	(0.76)
Deep-MLSA	−0.0004	−0.0006	−0.0003	−0.0008
	(−0.68)	(−0.87)	(−0.41)	(−1.30)
DeepMoji	0.0013	0.0001	−0.0007	−0.0002
	(1.75)	(0.17)	(−0.73)	(−0.26)

Panel B: StockTwits				
	$t+1$	$t+2$	$t+3$	$t+4$
Harvard-IV	**0.0028**	**0.0019**	**0.0019**	0.0011
	(4.44)	(2.69)	(2.42)	(1.44)
LM	**0.0034**	**0.0025**	0.0011	0.0012
	(5.42)	(4.18)	(1.50)	(1.60)
L1	**0.0022**	**0.0015**	0.0008	0.0006
	(2.79)	(1.99)	(0.96)	(0.71)
L2	**0.0024**	**0.0016**	0.0002	0.0001
	(3.37)	(2.26)	(0.35)	(0.09)
VADER	**0.0025**	**0.0019**	**0.0017**	0.0010
	(3.54)	(2.61)	(2.14)	(1.24)
Naive Bayes	0.0003	0.0001	−0.0001	−0.0007
	(0.34)	(0.08)	(−0.19)	(−0.98)
Max. entropy	−0.0002	−0.0002	−0.0012	−0.0007
	(−0.21)	(−0.21)	(−1.50)	(−0.96)
Deep-MLSA	0.0000	0.0003	−0.0011	−0.0015
	(−0.02)	(0.41)	(−1.76)	(−2.37)
DeepMoji	**0.0019**	0.0003	0.0007	0.0006
	(2.42)	(0.40)	(0.93)	(0.76)

Note: The table reports average cross-sectional regression coefficients (see Fama and MacBeth 1973) for daily bullish investor sentiment estimated from short messages published on Twitter (Panel A) and StockTwits (Panel B) mentioning a unique cashtag. The rows refer to the respective investor sentiment measure. The columns represent the dependent variable, being the *h*-day ahead retail investors’ order imbalance, for $h = 1, \dots , 4$. Newey-West (1987) standard errors are used to construct *t*-statistics, which are reported in parentheses below the respective coefficient estimate. All covariates are standardized such that the reported parameters can be interpreted as the effect of a one standard deviation change in that variable

**Table 9:** Differences in Fama-MacBeth (1973) regression coefficients for daily bullish sentiment (unique cashtags)

Panel A: Twitter									
	(1)	(2)	(3)	(4)	(5)	(6)	(7)	(8)	(9)
Harvard-IV (1)	–	**−2.27**	-0.39	−1.92	−0.06	**−2.15**	−1.62	1.67	−0.28
LM (2)		–	1.73	0.16	**2.18**	−0.25	0.39	**3.69**	1.75
L1 (3)			–	−1.92	0.30	**−2.15**	−1.36	**1.97**	0.10
L2 (4)				–	1.92	−0.45	0.27	**3.45**	1.94
VADER (5)					–	**−2.10**	−1.51	1.74	−0.23
Naive Bayes (6)						–	1.01	**3.98**	**2.26**
Max. entropy (7)							–	**3.17**	1.52
Deep-MLSA (8)								–	−1.86
DeepMoji (9)									–

Panel B: StockTwits									
	(1)	(2)	(3)	(4)	(5)	(6)	(7)	(8)	(9)
Harvard-IV (1)	–	−0.78	0.65	0.52	0.38	**2.95**	**3.30**	**3.41**	1.08
LM (2)		–	1.38	1.32	1.10	**3.70**	**4.09**	**4.37**	1.80
L1 (3)			–	−0.20	−0.31	**2.50**	**3.01**	**2.37**	0.41
L2 (4)				–	−0.15	**2.89**	**3.54**	**2.51**	0.64
VADER (5)					–	**2.56**	**2.95**	**2.98**	0.72
Naive Bayes (6)						–	0.90	0.28	**−2.27**
Max. entropy (7)							–	−0.15	**−2.84**
Deep-MLSA (8)								–	**−2.01**
DeepMoji (9)									–

Note: The table reports *t*-statistics for the difference in the average regression coefficients for daily bullish investor sentiment estimated from short messages published on Twitter (Panel A) and StockTwits (Panel B) with unique cashtags. The dependent variable of the cross-sectional regressions is the one-day ahead retail investors’ order imbalance. More precisely, we take the difference between coefficients obtained from methodologies reported in the rows with those reported in columns. The t-statistics are constructed using Newey-West (1987) standard errors. Differences which are statistically significant at the 5% level are highlighted by boldfaced numbers

The findings reported in Tables 6, 7, 8, 9 show that dictionaries tailored specifically towards financial topics, such as the L2 and LM dictionaries, are able to capture investor sentiment quite well—and in some cases even better than machine learning approaches. The results presented thus far focus on the predictive power of online investor sentiment for retail investors’ order imbalances. However, academics and practitioners alike are usually more interested in asset pricing implications. We address the effect of the nine sentiment estimation approaches on stock returns in the next section.

## Model-free forecasts of annualized abnormal portfolio returns

Initially, prior research has disregarded the role of irrational investors, assuming that arbitrageurs would trade against them and keep prices at their fundamental values (Friedman 1953; Fama 1965). More recent theoretical models and empirical findings suggest instead that arbitrageurs are likely to be risk-averse, and their willingness to trade against noise traders is limited (De Long et al. 1990; Shleifer and Vishny 1997). The model introduced by De Long et al. (1990), for instance, postulates that arbitrageurs face not only fundamental risks when taking positions against noise traders but also the risk that the beliefs of irrational investors may not reverse to their mean for a prolonged period of time. This implies that noise traders can drive stock prices away from their fundamental values, at least over short time periods, given that the willingness of risk-averse arbitrageurs to bet against them is limited. Following these theoretical postulations and corresponding empirical findings (e.g., Tetlock 2007; Baker and Wurgler 2006, 2007; Barber et al. 2009), we expect to observe a positive relation between a given measure of investor sentiment and future short-term returns.

Thus, we now investigate the ability of the different investor sentiment measures estimated from short messages published on Twitter and StockTwits to forecast annualized abnormal portfolio returns in a model-free setup (for a similar exercise focusing on online search intensity and a weekly trading pattern, see Joseph et al. 2011). To this end, denote by *q* the 10%-quantile and by $(1-q)$ the 90%-quantile of the empirical distribution of the respective investor sentiment measure across stocks on a given trading day.^24^ On each trading day, we form two equal-weighted portfolios of stocks based on the bullish sentiment of the previous trading day for each of the considered sentiment measures. The first portfolio (Short) contains the stocks for which the estimated online investor sentiment on the previous (trading) day is $\le q$. Conversely, the second portfolio (Long) contains the stocks for which the estimated online investor sentiment on the previous trading day is $\ge (1-q)$. A long-short raw portfolio return (Long – Short) is obtained as the difference between these two raw portfolio returns. The stocks are held in the portfolio for 1 trading day and are then re-sorted on the following trading day. Thus, we implement a daily sorting exercise of zero-cost portfolios.

Based on the assumption that a positive sentiment shock leads to an increase in returns and, conversely, a negative sentiment shock to a decrease in returns, the long-short portfolio return should yield a positive return across the considered sentiment measures. Our choice of *q* is guided by the aim to include only stocks exhibiting a rather extreme positive or negative sentiment on the previous trading day. Given the classification issues of some sentiment measures, as elaborated upon in Section 2, the two portfolios often contain more than 36 stocks per trading day. In terms of robustness, results remain unchanged qualitatively if we consider, for example, the first and last quintile for *q* and $(1-q)$, respectively. Since we want to investigate the forecasting performance of different investor sentiment measures in such a hypothetical and model-free portfolio trading application, transaction costs are ignored.

Abnormal, or risk-adjusted, portfolio returns are then obtained as follows: For each portfolio, we run a regression of daily excess returns on the three factors of Fama and French (1993) and the momentum factor of Carhart (1997), which have been found to explain cross-sectional differences in stock returns empirically. Thus, in each case, the regression is given by:

5 $$\begin{aligned} R_{p,t} - R_{f,t} = \alpha + \beta _m (R_{m,t} - R_{f,t}) + \beta _s \text {SMB}_{t} + \beta _h \text {HML}_{t} + \beta _m \text {MOM}_{t} + \varepsilon _t, \end{aligned}$$

where $R_{p,t}$ is the portfolio return on trading day *t*, $R_{f,t}$ is the risk-free rate, $(R_{m,t} - R_{f,t})$ denotes the excess return on the market, $\text {SMB}_{t}$ is the difference of returns between portfolios of “small” and “big” stocks, $\text {HML}_{t}$ refers to the return difference between portfolios consisting of “high” and “low” stocks as categorized by the book-to-market ratio, and $\text {MOM}_{t}$ denotes the momentum factor of Carhart (1997). Both data on the three factors of Fama and French (1993) and the momentum factor of Carhart (1997) are obtained from French’s website.^25^ Accordingly, the daily abnormal return is given by $\alpha$. As mentioned above, we report the implied annualized return for both raw and abnormal returns, the latter calculated as $(1 + \alpha )^{252}-1$, which denotes the total return from holding the portfolio for one year. Statistical inference is based on Newey-West (1987) standard errors and statistical significance at the 5% level is indicated by boldfaced numbers. Results for bullish Twitter and StockTwits sentiment are shown in Table 10.

**Table 10:** Annualized portfolio returns based on daily bullish sentiment

Panel A: Twitter						
	Raw return			Risk-adjusted return		
	Short	Long	Long-Short	Short	Long	Long-Short
Harvard-IV	**14.84**	**13.92**	−0.92	0.88	0.13	−0.90
	(3.18)	(2.88)	(−0.50)	(0.61)	(0.09)	(−0.51)
LM	**12.28**	**13.97**	1.69	−1.58	−0.06	1.37
	(2.37)	(3.11)	(0.82)	(−1.10)	(−0.05)	(0.70)
L1	**13.55**	**14.84**	1.29	−0.61	1.01	1.47
	(2.68)	(3.36)	(0.65)	(−0.38)	(0.67)	(0.77)
L2	**11.56**	**15.19**	3.62	−2.65	1.35	3.84
	(2.17)	(3.28)	(1.66)	(−1.59)	(0.87)	(1.89)
VADER	**14.82**	**14.89**	0.07	1.09	0.99	−0.26
	(2.95)	(3.02)	(0.04)	(0.77)	(0.65)	(−0.14)
Naive Bayes	**14.86**	**14.44**	−0.41	0.71	0.80	−0.05
	(2.90)	(3.16)	(−0.22)	(0.50)	(0.51)	(−0.03)
Max. entropy	**13.74**	**13.33**	−0.41	−0.41	−0.32	−0.06
	(2.72)	(2.82)	(−0.21)	(−0.28)	(−0.20)	(−0.03)
Deep-MLSA	**12.59**	**14.90**	2.31	−1.34	0.93	2.12
	(2.59)	(3.12)	(1.23)	(−0.97)	(0.74)	(1.16)
DeepMoji	**14.31**	**15.12**	0.81	0.33	1.15	0.66
	(2.98)	(3.22)	(0.43)	(0.24)	(0.78)	(0.35)

Panel B: StockTwits						
	Raw return			Risk-adjusted return		
	Short	Long	Long-Short	Short	Long	Long-Short
Harvard-IV	**14.37**	**13.51**	−0.87	0.26	−0.31	−0.73
	(3.01)	(2.90)	(−0.49)	(0.17)	(−0.24)	(−0.43)
LM	**11.23**	**14.20**	2.97	**−2.71**	0.35	2.91
	(2.17)	(3.15)	(1.63)	(−2.04)	(0.29)	(1.62)
L1	**12.32**	**14.72**	2.40	−2.04	0.83	2.72
	(2.58)	(3.16)	(1.31)	(−1.52)	(0.58)	(1.55)
L2	**12.06**	**18.23**	**6.18**	−1.94	**4.49**	**6.28**
	(2.51)	(3.84)	(2.99)	(−1.38)	(2.75)	(2.94)
VADER	**12.90**	**16.05**	3.14	−0.95	2.14	2.94
	(2.68)	(3.49)	(1.65)	(−0.66)	(1.59)	(1.61)
Naive Bayes	**12.81**	**14.91**	2.10	−1.49	1.58	2.92
	(2.63)	(3.39)	(1.10)	(−1.11)	(1.11)	(1.53)
Max. entropy	**13.73**	**15.16**	1.44	−0.53	1.64	2.02
	(2.83)	(3.24)	(0.73)	(−0.38)	(1.09)	(1.03)
Deep-MLSA	9.67	**14.32**	**4.65**	**−4.50**	0.52	**4.87**
	(1.88)	(3.05)	(2.36)	(−3.23)	(0.39)	(2.64)
DeepMoji	**13.92**	**16.75**	2.83	−0.27	**3.13**	3.25
	(2.87)	(3.71)	(1.55)	(−0.19)	(2.21)	(1.77)

Note: The table depicts the annualized raw and risk-adjusted returns (in %) of three portfolios based on daily bullish sentiment estimated from short messages published on Twitter (Panel A) and StockTwits (Panel B). The first portfolio (Short) contains the stocks for which the estimated online investor sentiment on the previous (trading) day is smaller than the 10% cross-sectional quantile. Conversely, the second portfolio (Long) contains the stocks for which the estimated online investor sentiment on the previous trading day is larger than the 90% cross-sectional quantile. A raw long-short portfolio return (Long-Short) is obtained as the difference between these two raw portfolio returns. The stocks are held in the portfolio for one trading day. The risk-adjusted returns are defined as the intercept of the regression of portfolio returns on the three risk factors introduced by Fama and French (1993) and the momentum factor of Carhart (1997). The *t*-statistics, reported in parenthesis, are constructed using Newey-West (1987) standard errors. Returns which are statistically significant at the 5% level are highlighted by boldfaced numbers

There are a few interesting findings: Firstly, looking at Panel A and the investor sentiment measures obtained from short messages published on Twitter, only the raw portfolio returns of the Short and Long portfolios are statistically significant at the 5% level. While not statistically significant, the long-short portfolio returns based on portfolios sorted according to investor sentiment estimated with Harvard-IV, naive Bayes, and maximum entropy are even negative. Secondly, looking at Panel B and the investor sentiment measures obtained from short messages published on StockTwits, we find statistically significant raw and risk-adjusted returns only for portfolios sorted based on the L2 dictionary and Deep-MLSA neural network. Interestingly, the long-short portfolio returns based on L2 are slightly larger than for Deep-MLSA. Again, the long-short portfolio returns based on portfolios sorted according to investor sentiment estimated with Harvard-IV are negative, albeit not statistically significant.

To asses whether the differences in raw and risk-adjusted annualized portfolio returns are statistically significant, we report *t*-statistics for the pairwise differences between long-short raw and risk-adjusted returns in Tables 11 and 12, respectively. The *t*-statistics reported are calculated for the difference between long-short returns obtained with the estimation method reported in the rows with that reported in the columns. More precisely, for each data source and each pair of sentiment estimation technique, we construct a time series of differences in the returns and compute *t*-statistics to test whether the average difference equals zero (using Newey-West (1987) standard errors). Panel A and B report the *t*-statistics for differences in returns using Twitter and StockTwits data, respectively. Differences which are statistically significant at the 5% level are highlighted by boldfaced numbers.

We especially consider the risk-adjusted returns from a portfolio sorting based on the empirical distribution of investor sentiment estimated from StockTwits data since these are statistically significant in some relevant cases. Most notably, while for risk-adjusted returns, the pairwise differences are statistically significant for Harvard-IV and L2 as well as for Harvard-IV and Deep-MLSA, the pairwise difference between L2 and Deep-MLSA is not statistically significant. Thus, the performance of the L2 dictionary and the Deep-MLSA neural network seems to be very similar in terms of their ability to predict annualized abnormal portfolio returns. This is still a striking finding since it shows that a dictionary, which is tailored well towards a specific kind of content, can at least compete with state-of-the-art machine learning based approaches. Thus, for practical applications in general, it might be worthwhile to consider building a dedicated dictionary for a specific type of textual data, instead of building a highly complex model that has to be trained on large amounts of labeled data before being of use.

**Table 11:** Differences in raw annualized returns of long-short portfolios

Panel A: Twitter									
	(1)	(2)	(3)	(4)	(5)	(6)	(7)	(8)	(9)
Harvard-IV (1)	–	−1.07	−0.97	−1.93	−0.47	−0.21	−0.22	−1.35	−0.76
LM (2)		–	0.16	−0.81	0.76	0.84	0.86	−0.26	0.35
L1 (3)			–	−1.29	0.55	0.84	0.80	−0.41	0.23
L2 (4)				–	1.56	1.89	1.84	0.52	1.32
VADER (5)					–	0.22	0.22	−0.94	−0.39
Naive Bayes (6)						–	0.00	−1.18	−0.61
Max. entropy (7)							–	−1.22	−0.59
Deep-MLSA (8)								–	0.59
DeepMoji (9)									–

Panel B: StockTwits									
	(1)	(2)	(3)	(4)	(5)	(6)	(7)	(8)	(9)
Harvard-IV (1)	–	−1.74	−1.49	**−3.27**	**−1.99**	−1.28	−1.03	**−2.37**	−1.71
LM (2)		–	0.26	−1.33	−0.07	0.36	0.62	−0.76	0.06
L1 (3)			–	−1.89	−0.33	0.14	0.46	−0.95	−0.21
L2 (4)				–	1.31	** 2.06**	** 2.31**	0.64	1.69
VADER (5)					–	0.50	0.80	−0.63	0.17
Naive Bayes (6)						–	0.42	−1.03	−0.44
Max. entropy (7)							–	−1.26	−0.71
Deep-MLSA (8)								–	0.77
DeepMoji (9)									–

Note: The table depicts the *t*-statistics of the average differences in raw returns of long-short portfolios based on daily bullish sentiment estimated from short messages published on Twitter (Panel A) and StockTwits (Panel B). More precisely, we take the difference between returns obtained from methodologies reported in the rows with those reported in columns. The *t*-statistics are constructed using Newey-West (1987) standard errors. Differences which are statistically significant at the 5% level are highlighted by boldfaced numbers

**Table 12:** Differences in risk-adjusted annualized returns of long-short portfolios

Panel A: Twitter									
	(1)	(2)	(3)	(4)	(5)	(6)	(7)	(8)	(9)
Harvard-IV (1)	–	−0.97	−1.08	**−2.09**	−0.31	−0.36	−0.38	−1.28	−0.69
LM (2)		–	−0.04	−1.04	0.78	0.59	0.59	−0.32	0.29
L1 (3)			–	−1.33	0.80	0.76	0.72	−0.27	0.39
L2 (4)				–	1.86	1.85	1.80	0.69	1.52
VADER (5)					–	−0.09	−0.09	−1.01	−0.48
Naive Bayes (6)						–	0.00	−0.95	−0.36
Max. entropy (7)							–	−0.97	−0.35
Deep-MLSA (8)								–	0.58
DeepMoji (9)									–

Panel B: StockTwits									
	(1)	(2)	(3)	(4)	(5)	(6)	(7)	(8)	(9)
Harvard-IV (1)	–	−1.68	−1.60	**−3.24**	−1.84	−1.61	−1.25	**−2.42**	−1.86
LM (2)		–	0.08	−1.40	−0.01	−0.01	0.36	−0.89	−0.15
L1 (3)			–	−1.77	−0.10	−0.09	0.34	−0.90	−0.26
L2 (4)				–	1.43	1.69	** 2.08**	0.60	1.52
VADER (5)					–	0.01	0.43	−0.81	−0.17
Naive Bayes (6)						–	0.57	−0.79	−0.20
Max. entropy (7)							–	−1.13	−0.63
Deep-MLSA (8)								–	0.69
DeepMoji (9)									–

Note: The table depicts the *t*-statistics of the average differences in risk-adjusted returns of long-short portfolios based on daily bullish sentiment estimated from short messages published on Twitter (Panel A) and StockTwits (Panel B). More precisely, we take the difference between returns obtained from methodologies reported in the rows with those reported in columns. Risk-adjusted returns are defined as the intercept plus the residuals of the regression in Equation (5). The *t*-statistics are constructed using Newey-West (1987) standard errors. Differences which are statistically significant at the 5% level are highlighted by boldfaced numbers

As a robustness check, we consider again the subsample of short messages published on Twitter and StockTwits that are identified by a unique cashtag. Results for bullish Twitter and StockTwits sentiment are shown in Table 13 and *t*-statistics for the pairwise differences between long-short raw and risk-adjusted returns in Tables 14 and 15, respectively. Although in this case, more of the long-short raw and risk-adjusted returns are statistically significant, the above findings do not change qualitatively. Interestingly, the portfolio returns based on a sorting according to investor sentiment estimated with DeepMoji are statistically significant now and very close to those based on Deep-MLSA. Overall, when predicting abnormal returns, considering short messages that can be identified with a unique cashtag seems to reduce noise and to improve performance quite a bit. Therefore, if return prediction is the goal, filtering short messages for unique cashtags should be considered.

**Table 13:** Annualized portfolios returns based on daily bullish sentiment (unique cashtags)

Panel A: Twitter						
	Raw return			Risk-adjusted return		
	Short	Long	Long-Short	Short	Long	Long-Short
Harvard-IV	**12.90**	**14.35**	1.45	−1.17	0.47	1.49
	(2.61)	(3.10)	(0.82)	(−0.91)	(0.35)	(0.86)
LM	**10.87**	**15.13**	**4.26**	**−2.99**	1.08	**3.92**
	(2.13)	(3.21)	(2.27)	(−2.06)	(0.82)	(2.24)
L1	**11.77**	**15.73**	**3.97**	−2.31	1.72	**3.88**
	(2.42)	(3.55)	(2.07)	(−1.55)	(1.19)	(2.10)
L2	**11.92**	**17.81**	**5.89**	−2.15	**3.92**	**5.92**
	(2.37)	(3.72)	(2.81)	(−1.56)	(2.74)	(3.04)
VADER	**13.19**	**15.91**	2.72	−0.59	1.99	2.42
	(2.66)	(3.36)	(1.49)	(−0.39)	(1.51)	(1.33)
Naive Bayes	**13.01**	**16.51**	3.50	−1.11	2.55	3.51
	(2.67)	(3.68)	(1.86)	(−0.79)	(1.64)	(1.89)
Max. entropy	**13.31**	**16.49**	3.18	−0.81	2.51	3.17
	(2.67)	(3.69)	(1.64)	(−0.59)	(1.57)	(1.69)
Deep-MLSA	**10.36**	**15.40**	**5.04**	**−3.64**	1.42	**4.91**
	(2.14)	(3.17)	(2.74)	(−2.72)	(1.14)	(2.81)
DeepMoji	**12.04**	**17.02**	**4.98**	−1.85	2.70	**4.40**
	(2.39)	(3.60)	(2.39)	(−1.21)	(1.70)	(2.18)

Panel B: StockTwits						
	Raw return			Risk-adjusted return		
	Short	Long	Long-Short	Short	Long	Long-Short
Harvard-IV	**12.30**	**16.04**	**3.74**	−1.62	2.04	**3.51**
	(2.56)	(3.43)	(2.42)	(−1.34)	(1.61)	(2.23)
LM	**11.50**	**13.97**	2.47	−2.51	−0.13	2.22
	(2.25)	(2.96)	(1.35)	(−1.92)	(−0.10)	(1.27)
L1	**10.47**	**14.79**	**4.32**	**−3.77**	1.11	**4.74**
	(2.23)	(3.16)	(2.40)	(−3.12)	(0.78)	(2.89)
L2	**11.49**	**18.25**	**6.77**	−2.52	**4.52**	**6.89**
	(2.37)	(3.77)	(3.67)	(−1.94)	(2.96)	(3.72)
VADER	**12.60**	**16.73**	**4.12**	−1.23	**2.63**	**3.71**
	(2.55)	(3.64)	(2.45)	(−1.01)	(2.10)	(2.22)
Naive Bayes	**12.80**	**15.51**	2.71	−1.41	1.86	3.12
	(2.73)	(3.35)	(1.60)	(−1.20)	(1.22)	(1.83)
Max. entropy	**12.59**	**15.35**	2.76	−1.57	1.75	3.17
	(2.65)	(3.36)	(1.64)	(−1.31)	(1.18)	(1.82)
Deep-MLSA	9.43	**15.15**	**5.71**	**−4.90**	1.22	**5.97**
	(1.81)	(3.30)	(2.62)	(−3.19)	(0.82)	(3.05)
DeepMoji	**10.96**	**16.51**	**5.55**	**−3.23**	2.58	**5.66**
	(2.32)	(3.53)	(3.06)	(−2.39)	(1.72)	(3.01)

Note: The table depicts the annualized raw and risk-adjusted returns (in %) of three portfolios based on daily bullish sentiment estimated from short messages published on Twitter (Panel A) and StockTwits (Panel B) mentioning a unique cashtag. The first portfolio (Short) contains the stocks for which the estimated online investor sentiment on the previous (trading) day is smaller than the 10% cross-sectional quantile. Conversely, the second portfolio (Long) contains the stocks for which the estimated online investor sentiment on the previous trading day is larger than the 90% cross-sectional quantile. A raw long-short portfolio return (Long-Short) is obtained as the difference between these two raw portfolio returns. The stocks are held in the portfolio for 1 trading day. The risk-adjusted returns are defined as the intercept of the regression of portfolio returns on the three risk factors introduced by Fama and French (1993) and the momentum factor of Carhart (1997). The *t*-statistics, reported in parenthesis, are constructed using Newey-West (1987) standard errors. Returns which are statistically significant at the 5% level are highlighted by boldfaced numbers

**Table 14:** Differences in raw annualized returns of long-short portfolios (unique cashtags)

Panel A: Twitter									
	(1)	(2)	(3)	(4)	(5)	(6)	(7)	(8)	(9)
Harvard-IV (1)	–	−1.30	−1.24	**−2.16**	−0.63	−1.01	−0.79	−1.45	−1.61
LM (2)		–	0.13	−0.73	0.72	0.34	0.46	−0.34	−0.29
L1 (3)			–	−1.04	0.59	0.24	0.39	−0.45	−0.49
L2 (4)				–	1.47	1.21	1.37	0.35	0.44
VADER (5)					–	−0.36	−0.21	−0.96	−1.00
Naive Bayes (6)						–	0.22	−0.63	−0.74
Max. entropy (7)							–	−0.74	−0.86
Deep-MLSA (8)								–	0.02
DeepMoji (9)									–

Panel B: StockTwits									
	(1)	(2)	(3)	(4)	(5)	(6)	(7)	(8)	(9)
Harvard-IV (1)	–	0.60	−0.31	−1.47	−0.21	0.48	0.45	−0.79	−0.81
LM (2)		–	−0.88	**−2.05**	−0.82	−0.11	−0.13	−1.38	−1.29
L1 (3)			–	−1.31	0.09	0.83	0.82	−0.54	−0.61
L2 (4)				–	1.22	** 2.17**	** 2.31**	0.43	0.66
VADER (5)					–	0.66	0.65	−0.69	−0.65
Naive Bayes (6)						–	−0.04	−1.28	−1.68
Max. entropy (7)							–	−1.31	−1.76
Deep-MLSA (8)								–	0.07
DeepMoji (9)									–

The table depicts the *t*-statistics of the average differences in raw returns of long-short portfolios based on daily bullish sentiment estimated from short messages published on Twitter (Panel A) and StockTwits (Panel B) mentioning a unique cashtag. More precisely, we take the difference between returns obtained from methodologies reported in the rows with those reported in columns. The *t*-statistics are constructed using Newey-West (1987) standard errors. Differences which are statistically significant at the 5% level are highlighted by boldfaced numbers

**Table 15:** Differences in risk-adjusted annualized returns of long-short portfolios (unique cashtags)

Panel A: Twitter									
	(1)	(2)	(3)	(4)	(5)	(6)	(7)	(8)	(9)
Harvard-IV (1)	–	−1.14	−1.19	**−2.20**	−0.46	−1.00	−0.77	−1.39	−1.34
LM (2)		–	0.02	−0.91	0.71	0.18	0.31	−0.44	−0.20
L1 (3)			–	−1.09	0.70	0.19	0.35	−0.43	−0.25
L2 (4)				–	1.63	1.22	1.38	0.41	0.74
VADER (5)					–	−0.51	−0.35	−1.06	−0.88
Naive Bayes (6)						–	0.23	−0.58	−0.44
Max. entropy (7)							–	−0.69	−0.59
Deep-MLSA (8)								–	0.19
DeepMoji (9)									–

Panel B: StockTwits									
	(1)	(2)	(3)	(4)	(5)	(6)	(7)	(8)	(9)
Harvard-IV (1)	–	0.62	−0.66	−1.65	−0.11	0.18	0.16	−1.01	−0.96
LM (2)		–	−1.14	**−2.24**	−0.76	−0.43	−0.43	−1.60	−1.46
L1 (3)			–	−1.16	0.48	0.84	0.82	−0.48	−0.46
L2 (4)				–	1.47	** 2.04**	** 2.15**	0.38	0.67
VADER (5)					–	0.29	0.26	−0.99	−0.90
Naive Bayes (6)						–	−0.04	−1.26	−1.51
Max. entropy (7)							–	−1.26	−1.57
Deep-MLSA (8)								–	0.13
DeepMoji (9)									–

The table depicts the *t*-statistics of the average differences in risk-adjusted returns of long-short portfolios based on daily bullish sentiment estimated from short messages published on Twitter (Panel A) and StockTwits (Panel B) mentioning a unique cashtag. More precisely, we take the difference between returns obtained from methodologies reported in the rows with those reported in columns. Risk-adjusted returns are defined as the intercept plus the residuals of the regression in Equation (5). The *t*-statistics are constructed using Newey-West (1987) standard errors. Differences which are statistically significant at the 5% level are highlighted by boldfaced numbers

## Conclusion

We have taken a pragmatic approach to answering the question of how to best gauge investor behavior by means of different online investor sentiment measures. Given the increasing number of publicly available dictionaries and implemented machine learning techniques that researchers and practitioners can use, our comparison of sentiment measures is restricted mostly to such publicly available approaches. The empirical analysis is based mainly on two financial applications that reveal the effects of the online investor measures on the cross-section of stocks—both in terms of retail investors’ order imbalances and forecasts of portfolio returns.

The performance of the considered sentiment measures varies considerably. We find the LM and L2 dictionaries to perform best throughout both applications. This finding is especially striking since the dictionary of Loughran and McDonald (2011) is not optimized for short messages published on social media platforms. These results demonstrate that publicly available dictionaries do not just constitute a methodology that ensures reproducibility of results but also that finance-specific dictionaries are at least on par or even superior to publicly available neural network techniques, such as the Deep-MLSA and DeepMoji, in financial applications. Thus, for future research, we strongly advocate the development of new and the refinement of existing dictionaries that not only cover specifics of financial terms but are also optimized for short messages published on social media platforms. The dictionaries of Renault (2017) may be taken as good examples. On a different note, publicly available machine learning techniques are still scarce. Our understanding of sentiment-driven investor behavior would benefit from researchers making their approaches available to others. Lastly, as our analyses demonstrate, empirical results involving online investor sentiment should always be scrutinized and compared with other approaches to the estimation of investor sentiment from online sources to avoid misleading conclusions.

## Footnotes

1 Note the similarity between noise trader and liquidity trader theories as elaborated upon in Tetlock (2007).

2 There also exists an extensive literature concerned with the influence of investor sentiment extracted from traditional news channels on financial market outcomes (e.g., Tetlock 2007; Garcia 2013; Hillert et al. 2014). Moreover, closely related to the literature on online investor sentiment is another strand of literature focusing on investor attention, measured by online search queries, and its usefulness in forecasting returns, volatility, and trading volume (e.g., Da et al. 2011; Joseph et al. 2011; Dimpfl and Jank 2016). Note, however, that this paper exclusively deals with investor sentiment derived from social media platforms.

3 See https://caps.fool.com/ for further information about CAPS.

4 Since Nov 7, 2017, the limit has been increased to 280 characters.

5 Note that in total 368 companies are included continuously in the S&P 500 between January 2011 and December 2017. Due to data issues, we have excluded eight companies from our data set.

6 We have also conducted our analysis using a more restrictive filter, i.e., considering only messages shared on Twitter or StockTwits mentioning a unique cashtag.

7 Between 2011 and 2017, 41 out of the 360 considered companies have either changed their name or their ticker.

8 For more information, see http://www.wjh.harvard.edu/~inquirer/homecat.htm.

9 See https://sraf.nd.edu/.

10 The Python implementation is available at https://github.com/cjhutto/vaderSentiment.

11 Note that the dictionaries introduced by Renault (2017) are constructed using a sample of short messages published on StockTwits between June 2013 and August 2014. Since this time period partially overlaps with our data set, we have conducted robustness checks of our results by removing observations between June 2013 and 2014. Our overall results remain unaffected. Detailed robustness checks are available upon request.

12 The dictionaries are available at http://www.thomas-renault.com/data.php.

13 For an overview of the naive Bayes classifier and other classification approaches used for finance-related text data, see Das et al. (2014).

14 The pre-trained Deep-MLSA model can be obtained from https://github.com/spinningbytes/deep-mlsa.

15 The pre-trained DeepMoji model can be obtained from https://github.com/bfelbo/DeepMoji.

16 We train the model over the same time period considered by Renault (2017) to construct the L1 and L2 dictionaries. This choice facilitates the comparison of the sentiment estimation approaches. In particular, we have conducted robustness checks of our results by removing observations between June 2013 and 2014. Our overall results remain unaffected. Detailed robustness checks are available upon request.

17 The fine-tuning of the pre-trained DeepMoji model is done by extending the word embedding with 10,000 additional word-tokens, replacing the output-layer with a sigmoid activation function and training the entire model using the “chain-thaw” approach (for more details, see Felbo et al. 2017).

18 As noted by Renault (2017), most stop words might contain relevant information in the context of the stock market (e.g., “up”). Therefore, we only remove the stop-words “a”, “an”, and “the”.

19 As a robustness check, instead of using the TF-IDF, we considered also a simple term frequency scheme. The general results remain unaffected.

20 As an alternative, we have conducted our analysis by replacing missing values with neutral sentiment, i.e., $B_{i,t}=0$. Our results remain unchanged qualitatively.

21 The first trading day of each new trading week constitutes a particular case since the short messages published on weekends have to be taken into account. Thus, in this case, we regress the first trading day’s order imbalance on the average of the daily sentiment from the last trading day of the previous trading week until the day prior to the first trading day of the new trading week. Moreover, if there is a holiday or other non-trading day during a given trading week, the sentiment of that day is used in the regression of the following trading day’s excess return.

22 The realized volatility is computed as the square-root of the sum of squared five minute log intraday returns. Intraday returns are calculated using transaction data obtained from the TAQ database and cleaned following the approach outlined in Barndorff-Nielsen et al. (2009).

23 The estimated coefficients for the control variables are available from the authors upon request.

24 Again, we take the investor sentiment on weekends and holidays into account in the same way as stated in the previous section.

25 See https://mba.tuck.dartmouth.edu/pages/faculty/ken.french/data_library.html.
